# Supplementary material for: Prognostic, Immunological, and Mutational Analysis of MTA2 in Pan-Cancer and Drug Screening for Hepatocellular Carcinoma
Source: Biomolecules. 2023 May 24;13(6):883. doi: 10.3390/biom13060883 (PMC10296612; doi:10.3390/biom13060883)
Supplement: Supplementary file 1 [file biomolecules-13-00883-s001.zip › biomolecules-2306571-supplementary.pdf]

Supplementary Material

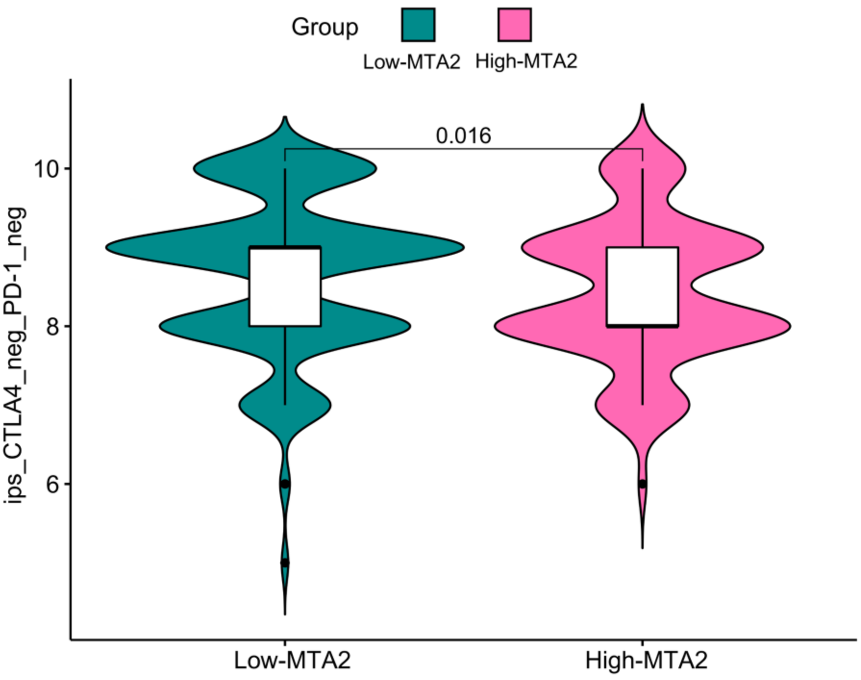

Figure S1. Immunophenoscore (IPS) showed higher scores in the high-MTA2 group ($p < 0.05$).

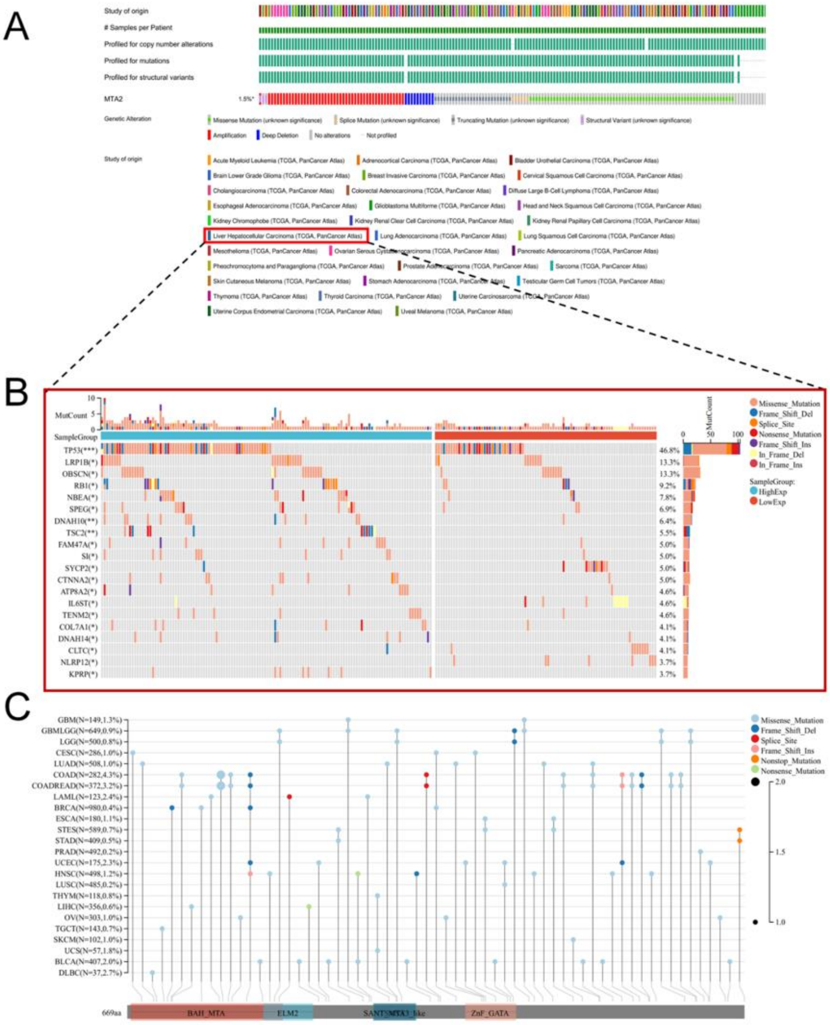

Figure S2. Mutation frequency of MTA2 in pan-cancer and mutations in high and low MTA2 expression groups in LIHC. (A) The mutation frequency of MTA2 in pan-cancer was 1.5% from cBioPortal database. (B) Mutational landscape of high-MTA2 and low-MTA2 groups in LIHC. (C) Mutation sites and mutation frequency of MTA2 in pan-cancer. *$p < 0.05$, **$p < 0.01$, ***$p < 0.001$.
